# Dynamic balance decreased in postdromal migraineurs compared to non migraine controls

**DOI:** 10.1186/1129-2377-14-S1-P73

**Published:** 2013-02-21

**Authors:** MT Moore, R Feuerherd

**Affiliations:** 1 Northern Michigan University, USA

## Introduction

Balance is a complex process involving visual, vestibular and neuromuscular control. Migraineurs often report vertigo and dizziness symptoms during and post migraine. The Biodex Balance System SD 1 is a reliable method to measure dynamic balance. Little research 2 has examined dynamic balance in migraineurs compared to individuals who do not have migraines.

## Purpose

The purpose of this research is to examine the differences between migraineurs and controls dynamic balance at two testing intervals: baseline (migraine free 7 days) and post migraine (within 48 hours of migraine onset).

## Methods

20 controls (C) (age 25.70± 10.77) and 17 Migraineurs (M) (age 25.24± 9.55) completed dual limb support testing on the Biodex Balance System SD. Limits of Stability (LOS) testing at moderate skill level (75%) involved center of gravity control within their base of support. The clinical test of sensory integration and balance tested stability and sway indexes within four conditions (eyes open/closed on firm vs foam surface) for 30 second intervals.

## Results

A repeated measures ANOVA revealed significant differences [mean diff (post-pre) C=7.06±5.57, M=2.68±7.45, p=.029] between migraineurs and non-migraineurs in overall LOS post migraine. Significant decreases were found between shift of balance to the right [mean diff (post-pre) C=14.71±17.69,M=-2.18±16.97, p=.034] and balance to the left [mean diff (post-pre) C=9.88±15.77, M= -3.75± 22.1, p=.019] post migraine. No significant differences were found between groups for overall LOS at baseline (p=.703). No significant differences were found between groups for stability or sway indexes for all conditions on the clinical test of sensory integration.

## Conclusions

Migraineurs exhibit difficulty with center of gravity shifts to the right and left and overall dynamic LOS post migraine. Once LOS is exceeded a fall, stumble or step will ensue. This suggests decreases in lower extremity strength, proprioception and vestibular deficiencies.
